# Clinical significance of serological biomarkers and neuropsychological performances in patients with temporal lobe epilepsy

**DOI:** 10.1186/1471-2377-12-15

**Published:** 2012-03-14

**Authors:** Chiung-Chih Chang, Chun-Chung Lui, Chen-Chang Lee, Shang-Der Chen, Wen-Neng Chang, Cheng-Hsien Lu, Nai-Ching Chen, Alice Y W Chang, Samuel H H Chan, Yao-Chung Chuang

**Affiliations:** 1 Department of Neurology, Kaohsiung Chang Gung Memorial Hospital and Chang Gung University College of Medicine, Kaohsiung, Taiwan; 2 Center for Translational Research in Biomedical Sciences, Kaohsiung Chang Gung Memorial Hospital and Chang Gung University College of Medicine, Kaohsiung, Taiwan; 3 Department of Radiology, Kaohsiung Chang Gung Memorial Hospital and Chang Gung University College of Medicine, Kaohsiung, Taiwan; 4 Department of Biological Science, National Sun Yet-sen University, Kaohsiung, Taiwan

**Keywords:** Biomarkers, Neuropsychological performances, Temporal lobe epilepsy, Gray matter atrophy, Heat shock proteins

## Abstract

**Background:**

Temporal lobe epilepsy (TLE) is a common form of focal epilepsy. Serum biomarkers to predict cognitive performance in TLE patients without psychiatric comorbidities and the link with gray matter (GM) atrophy have not been fully explored.

**Methods:**

Thirty-four patients with TLE and 34 sex - and age-matched controls were enrolled for standardized cognitive tests, neuroimaging studies as well as measurements of serum levels of heat shock protein 70 (HSP70), S100ß protein (S100ßP), neuronal specific enolase (NSE), plasma nuclear and mitochondrial DNA levels.

**Results:**

Compared with the controls, the patients with TLE had poorer cognitive performances and higher HSP70 and S100ßP levels (*p *< 0.01). The patients with higher frequencies of seizures had higher levels of HSP70, NSE and S100ßP (*p *< 0.01). Serum HSP70 level correlated positively with duration of epilepsy (σ = 0.413, *p *< 0.01), and inversely with memory scores in the late registration (σ = −0.276, *p *= 0.01) and early recall score (σ = −0.304, *p *= 0.007). Compared with the controls, gray matter atrophy in the hippocampal and parahippocampal areas, putamen, thalamus and supplementary motor areas were found in the patient group. The HSP70 levels showed an inverse correlation with hippocampal volume (R square = 0.22, *p *= 0.007) after controlling for the effect of age.

**Conclusions:**

Our results suggest that serum biomarkers were predictive of higher frequencies of seizures in the TLE group. HSP70 may be considered to be a stress biomarker in patients with TLE in that it correlated inversely with memory scores and hippocampal volume. In addition, the symmetric extratemporal atrophic patterns may be related to damage of neuronal networks and epileptogenesis in TLE.

## Background

Temporal lobe epilepsy (TLE) represents the most common and likely to be medically refractory focal epilepsy in adults, with an estimated prevalence of 40% among epilepsy patients [1]. Although epileptic focus in most cases of TLE originates from the temporal lobe and particularly the hippocampus, virtually all patients develop complex partial seizures with or without secondary generalization [2]. Antiepileptic drugs (AEDs) are usually successful in controlling secondarily generalized seizures, however as many as 30% to 40% of TLE patients still have seizures despite receiving appropriate medical management [3]. Those with pharmacoresistant TLE often require surgical treatment [4] along with lifelong AED therapy, and are associated with greater comorbidities and functional disabilities [5].

Proper selection of serological biomarkers, for example prolactin [6], can extend the diagnostic repertoire of epileptic seizures in the clinical setting. There were studies exploring the relationship between serum S100ß protein (S100ßP), neuron specific enolase (NSE) and heat shock protein 70 (HSP70) in Alzheimer dementia [7], status epilepticus [8] and epilepsy syndrome [9]. Upregulation of HSP70 protects against intracellular amyloid toxicity by rescuing proteasomal activity [7]. Pathology study in sudden and unexpected death in epilepsy suggested that HSP70 overexpression indicates acute neuronal injury occurring in the ante-mortem stage [9]. Elevated serum NSE has been reported in patients with status epilepticus, complex partial status, in addition to TLE [8,10,11]. In TLE, a few studies have suggested that serum S100ßP [12,13], NSE [14] or HSP70 [15-17] may be useful biomarkers for central nervous system damage. However, little data exists with regards to these biomarkers and cognitive performances in TLE. Elevation of plasma nuclear and mitochondrial DNA has been considered to be the consequence of neuronal cell death in the brain parenchyma, and has been linked with poor outcomes in bacterial meningitis and ischemic stroke [18,19]. However, whether these two biomarkers are also associated with poor cognitive performances in TLE has not been reported before.

TLE can be a progressive disorder, potentially resulting in structural damage and a decline of cognitive abilities over time [20]. Impairment in cognitive performance beyond the memory domain has been frequently reported in patients with TLE [21,22], and a link between cognitive deficits with structural changes has been proposed. One meta-analysis reviewed the voxel-based morphometry (VBM) results in patients with TLE [23] and showed extratemporal gray matter (GM) atrophy. Although GM atrophy in TLE has been reported to be greater on the ipsilateral side in terms of epileptogenesis, the correlation accounting for cognitive deficits and the relationship with biomarkers have not been fully explored.

Recent studies have highlighted that self-propagating seizure activities in TLE may result in dysregulated inflammation, blood brain barrier damage and neuronal damage [24]. Since HSP70, S100ßP, NSE, as well as plasma nuclear and mitochondrial DNA levels have been shown to reflect central nervous system damage, the present study hypothesized that these biomarkers may be of prognostic value in TLE patients from the cognitive aspect. We attempted to validate our hypothesis by measuring these biomarkers in TLE patients compared with age-and sex-matched controls, and compared the cognitive performances and structural imaging analysis using VBM in the GM areas. Furthermore, correlations between the biomarkers with duration of epilepsy, frequency of seizures and GM volume in the TLE group were examined in an attempt to understand the relationship between these biomarkers in predicting the severity of TLE and related neuronal damage.

## Methods

### Study design

This was a single center, age-and sex-matched cross-sectional study which was approved by the Institutional Review Board of Chang Gung Memorial Hospital and complied with the ethical standards established in the Declaration of Helsinki. The experiments were undertaken with the written, informed consent of each subject and their caregiver (when appropriate).

### Patient enrolment

This study was initiated at the epilepsy outpatient clinic of Kaohsiung Chang Gung Memorial Hospital in 2009. Patients followed up as the epilepsy cohort [25] all underwent an extensive investigation including clinical history, comprehensive neurologic examination, interictal EEG and routine visual MRI analysis, the protocol of which included T1 and T2 sequences in axial and coronal planes.

The clinical diagnosis of TLE was based on the International League Against Epilepsy criteria (1997) as follows: (1) seizure semiology consistent with TLE, with abdominal, epigastric, psychic, or autonomic auras, followed by behavioral arrest, progressive alteration of consciousness, oroalimentary, and manual automatisms; (2) mesial and/or anterior temporal interictal spikes from video-electroencephalography (EEG) or bilateral sphenoidal EEG; and (3) no lesions other than increased T2 signal and/or atrophy in hippocampal formation identified by MRI.

Because it was not possible to combine all the influential factors in the TLE group to produce a uniform population, we only included non-surgical patients. By family history and past medical history review, none of our study patient had family trait or childhood febrile seizure history. Additional exclusion criteria in this study included a known history of mental retardation and a psychiatric comorbidity that prevented either a neuropsychiatric interview or neuroimaging. We also excluded patients with any of the following: (1) medication history of psychoactive or central nervous system depressant drugs; and (2) abnormal liver or renal functions. These exclusion criteria were added to avoid the confounding effects of medication and physical disorders on the cognitive test results.

After screening our TLE cohort [25], 34 patients (15 males and 19 females) fulfilled the inclusion and exclusion criteria, agreed to participate in the study, and completed it. Data for the age at onset, duration of epilepsy, average seizure frequency per month during the previous year, and numbers of AED were analyzed. According to the seizure frequency, patients with two or fewer seizures per month were classified as Group 1, and patients with more than two seizures per month as Group 2. Furthermore, 34 age-and sex-matched healthy subjects from the normative database were used as controls for the biomarkers, neuropsychological testing and MRI comparison. The age of each control subject was within 1 year of the matched TLE patient. None of the control subjects had a history of neurologic or neuropsychiatric disorders, and all had normal MRI and basic blood test results (liver and renal function tests, electrolytes, and complete blood cell counts).

### Analysis of serological biomarkers

Blood samples were taken between 8 and 10 am after overnight fasting for the analysis of serum levels of HSP70, NSE and S100ßP. Liver and renal function tests, electrolytes, and complete blood cell counts were also analyzed by the central laboratory of Chang Gung Memorial Hospital-Kaohsiung.

For each patient, 5 ml of peripheral venous blood was collected into a serum separating tube. Plasma was prepared using EDTA-containing tubes and centrifugation at 3,000 rpm for 10 minutes, isolated, and immediately stored at −80 C in multiple aliquots. Serum HSP70, NSE and S100ßP levels were measured using an enzyme-linked immunosorbent assay (ELISA) kit (HSP70: EKS-750, Assay Design, San Francisco, CA, USA.; NSE: TM E-4700, Labor Diagnostika Nord, Nordhorn, Germany; S100ßP: RD192090100R, BioVendor, Brno, Czech Republic). In the ELISA, the samples were incubated in multiwell plates that had been coated with markers specific for anti-HSP70, anti-NSE, and anti-S100ßP antibodies. The ELISA procedures followed the manufacturer's protocols. The degrees of enzymatic turnover of the substrate were determined by dual wavelength absorbance measurements at 450 nm using a multiscan spectrum reader (Thermo Scientific, Miami, FL, USA). The antigen standards were used to plot a standard curve of absorbance versus antigen concentration from which the antigen concentrations in the unknowns were calculated.

### Analysis of plasma nuclear and mitochondrial DNA levels

To evaluate the neuronal cell damage, we detected the released specific mitochondrial DNA (ND2 gene) and nuclear DNA (beta globulin gene) levels in plasma by real-time quantitative PCR methods [19,26,27]. The analysis of plasma nuclear and mitochondrial DNA levels followed our previous methods [18,19]. In brief, 5 ml of peripheral venous blood in an EDTA-containing tube was collected and the blood sample was centrifuged at 3000 rpm for 10 minutes. 400 µl of plasma sample extracted by a QIAamp DNA Mini Blood Kit (No. 51304, Qiagen, Düsseldorf, Germany) was used for each measurement. Primers of the beta globulin gene (forward: 5'-GTG CAC CTG ACT CCT GAG GAG A-3'; reverse: 5'-CCT TGA TAC CAA CCT GCC CAG-3') and ND2 gene (forward: 5'-CAC AGA AGC TGC CAT CAA GTA-3'; reverse: 5'-CCG GAG AGT ATA TTG TTG AAG AG-3') were used, and the concentration adjusted to 10 µM containing 0.2 µl of each primer, 10 µl 2X SYBR Green PCR Fast Master Mix (No. 4385612, Applied Biosystems, San Francisco, CA, USA), 7.6 µl H2O and 2 µl plasma DNA sample. The mixture was placed in a StepOnePlus real-time PCR system (Applied Biosystems) for reaction following the standardized PCR procedure for 40 cycles: 95 C for 20 seconds, followed by 95 C for 3 seconds and 60 C for 30 seconds. Quantification of the PCR results followed the Pfaffl method[28].

### Cognitive and behavior tests

All of the cognitive tests were performed during the interictal state as evidenced by the EEG recording before the cognitive test. A clinical neuropsychologist blinded to the patients' clinical diagnosis performed the neuropsychological tests which were chosen to assess memory, executive, attention, and visuospatial functions [29-31]. Verbal and visual episodic memory were assessed by a modified California Verbal Learning Test-Mental Status [32] and the Rey-Osterrieth Complex figure after a 10-minute delay. Language screening included the 16-item Boston Naming Test and 3-step comprehension and semantic verbal fluency.

Visuo-spatial abilities were assessed by a modified Rey-Osterrieth Complex Figure, pentagons, a transparent cube copy, and by the number location test from the Visual Object and Space Perception Battery. The ability to perform five arithmetic calculations was assessed, while frontal lobe function was assessed by digit forward and backward span, design fluency, Stroop Interference test, and the Modified Trails B test. Neuropsychiatric inventory (NPI) was used to assess behavioral symptoms. All of the tests were administered to both the patients and the controls for a statistical comparison of non-standardized tests.

### MRI protocols and VBM

MRI was performed using a 3.0 Tesla scanner (Excite, GE Medical System, Milwaukee, WI, USA) equipped with echo-planar capability. Three-dimensional spoiled gradient-recalled acquisition in a steady state sequence was performed with the following parameters: TR/TI = 8600 ms/400 ms, FOV: 240 mm × 240 mm, slice thickness 1 mm.

VBM was processed using the SPM5 software package (http://www.fil.ion.ucl.ac.uk/spm) with study specific templates [30]. All T1-weighted images were spatially normalized into the standardized Montreal Neurological Institute space using a 12-parameter affine transformation and non-linear normalization. The study-specific template was then smoothed with an 8 mm full-width at half-maximum (FWHM) isotropic Gaussian kernel.

The original images were warped to match the customized templates and re-sliced onto a voxel size of 1 × 1 × 1 mm to minimize partial volume effects. The images were segmented into gray, white, and cerebrospinal fluid (CSF) compartments with intensity inhomogeneity correction, modulated with Jacobian determinants to compensate for volume changes in non-linear spatial normalization, and smoothed with a 10 × 10 × 10 mm FWHM isotropic Gaussian Kernel [33]. A general linear model was used to assess statistical differences in GM.

### Statistical analysis

Categorical variables were compared using the chi-square test. Neuropsychiatric performances between the two groups were analyzed by the Mann-Whitney *U *test with Bonferroni correction. Partial correlation analysis between duration of epilepsy and serum biomarker/neuropsychological performances was performed with age being considered as a confounding factor. Multiple linear regression analysis was used to examine the relationship between GM atrophy and serum biomarkers levels. All statistical analyses were performed using the SAS software package (version 9.2, SAS Statistical Institute, Cary, NC, USA). A p value < 0.01 was considered statistically significant.

For VBM analysis, a significance threshold was set at p < 0.001 and corrected for multiple comparisons across the whole brain (false discovery rate) with an extended threshold of 250 voxels and applied to the resulting *t*-statistic maps of GM. To detect any correlation of clinical data with GM atrophy, partial correlation analysis of GM partitions was applied to the biomarkers adjusted for the effect of age at a significance of p < 0.01, corrected for multiple comparisons.

## Results

### Demographic data and clinical characteristics of the patients with TLE

The age of the patient group was 37.1 ± 10.3 years, which was not significantly different from the control group (37.2 ± 10.2 years). Educational levels were not significantly different between the patient group (11.5 ± 4.3 years) and the controls (12.2 ± 3.4 years). The age of onset in the patient group was 18.8 ± 12.7 years and the duration of epilepsy was 18.9 ± 11.9 years. According to seizure frequency defined above, 22 patients were classified as Group 1 (0.8 ± 0.3 times per month), and 12 patients as Group 2 (3.3 ± 0.9 times per month). Eight patients received monotherapy, and the remaining 26 patients received polytherapy including 19 patients with two AEDs and 7 patients with 3 AEDs. Among the patients with epileptic focus, 21 had left temporal origins, and 13 from the right side. According to semiology, 9 patients experienced complex partial seizures only, while the other 25 patients had partial seizures with or without secondary generalization. Two patients had one or more seizures despite being treated with two consecutive first-line antiepileptic medications.

### Biomarkers and cognitive tests

Table 1 shows the results of biomarkers and cognitive tests in patients with TLE and the control group. While all of the five selected biomarkers were higher in the patient group compared with the controls, only levels of HSP70 and S100ßP reached statistical significance (p < 0.001). A comparison of biomarker levels between those with initial left versus right epileptogenicity showed no statistical significance.

**Table 1:** Biomarkers and neuropsychiatric performances between patients with temporal lobe

	Controls (n = 34)	TLE patients (n = 34)
Biomarkers		
Heat shock protein 70 (μg/ml)	130.84 ± 61.1	194.5 ± 116.1**
Non-specific enolase (ng/ml)	9.43 ± 1.9	9.51 ± 1.9
S100ß protein (pg/ml)	70.52 ± 28.0	99.83 ± 45.7 **
Plasma nuclear DNA (ng/ml)	28.62 ± 2.5	29.19 ± 2.7
Plasma mitochondrial DNA (ng/ml)	21.33 ± 1.1	21.15 ± 1.0
Verbal Memory CVLT-MS (9)		
T1	6.18 ± 1.1	5.18 ± 1.5**
T2	7.74 ± 1.1	7.36 ± 1.4
T3	8.24 ± 0.9	8.06 ± 1.2
T4	8.59 ± 0.7	8.15 ± 1.0
30 sec free recall	8.65 ± 0.7	7.67 ± 2.0**
10-min free recall	8.38 ± 1.2	7.18 ± 2.3**
10-min recognition	8.53 ± 0.9	7.55 ± 1.8
Visual Memory		
Modified Rey-Osterrieth recall (17)	15 ± 2.5	11.64 ± 4.5**
Visuospatial Functions		
Modified Rey-Osterrieth copy (17)	17 ± 0	16.79 ± 0.7
Cube copy (2)	1.82 ± 0.5	1.05 ± 0.9
Pentagon copy (1)	1 ± 0	0.82 ± 0.4
Visual object and space perception (10)	8.29 ± 2.0	8.64 ± 1.8
Speech and Language Ability		
Semantic Fluency (1 minute)		
Animal	21.59 ± 3.9	15.3 ± 5.4**
Fruit	15.75 ± 2.8	11.54 ± 3.4**
Transportation	13.83 ± 3.8	9.26 ± 2.1**
Town	24.28 ± 6.2	16.82 ± 6.2**
Boston naming test (16)	15.58 ± 0.8	14.36 ± 2.2**
Comprehension (4)	3.88 ± 0.3	3.51 ± 0.9
Abstract thinking (3)	2.58 ± 0.7	1.97 ± 1.2
Problem solving (3)	2.60 ± 0.7	2.00 ± 1.1
Executive function		
Digit backward	5.0 ± 1.6	4.74 ±1.5
Stroop interference correct (1 minute)	54.1 ± 10.9	40.33 ± 14.6**
Design fluency	10.78 ± 3.0	8.56 ± 5.6*
Trail Making test time (< 120 seconds)	23.6 ± 11.6	47.8 ± 35.3**
Correct line in Trail Making (14)	13.13 ± 2.7	11.92 ± 3.5
Calculation (5)	4.75 ± 0.7	4.51 ± 0.8
Digit forward	8.40 ± 0.8	7.44 ± 1.4**
Neuropsychiatric inventory score	1.91 ± 6.6	2.84 ± 5.5

Values expressed as mean ± standard deviation.

Number in parenthesis following task name = maximal scores

**p < 0.01. *p < 0.05 between TLE patients and controls

*TLE *temporal lobe epilepsy; *CVLT-MS *California Verbal Learning Test-Mental Status

In the selected cognitive tests, the short and long delayed verbal memory, visual memory, speech and language ability, executive function tests and digit forward spans were all significantly lower in the patient group than in the control group (all p < 0.01). There was no statistical difference in NPI total score between the patients and the controls.

### Correlation study between biomarkers with cognitive tests

In the patient group, the correlation between age and duration of epilepsy was not significant (σ = 0.313, *p *= 0.07). Decreased memory scores in the CVLT-MS late registration (σ = −0.276, *p *= 0.01) and early recall score (σ = −0.304, *p *= 0.007) were inversely associated with serum HSP70 level. The level of plasma nuclear DNA showed an inverse correlation with verbal fluency (σ = −0.297, *p *= 0.008). A positive correlation was found between plasma nuclear DNA and Trail Making test completion time (σ = 0.333, *p *= 0.003), however after enrolling the duration of epilepsy as a covariate into the regression model, the correlation was not significant.

### GM atrophy and regional variability

**Figure 1:**
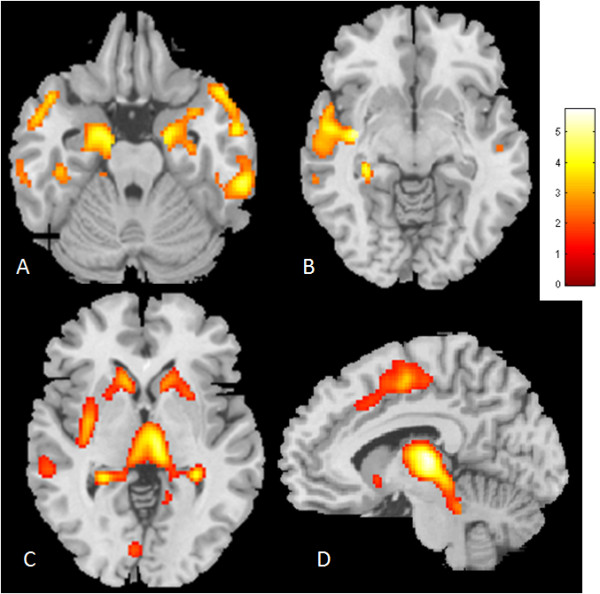
**Voxel based morphometry revealed atrophy regions in the temporal lobes of epilepsy patients in a wide range of brain areas as compared with the age-matched controls**. **A**. Hippocampus and lateral temporal regions; **B**. Upper temporal and tail of the hippocampus; **C**. Striatum and thalamus; **D**. Supplementary motor area. The anatomical reference = Montreal Neurological Institute Template.

When all TLE patients were compared with the controls, wide brain regions on the striatum, frontal and temporal lobes were significantly different (Table 2, Figure 1). In addition to the involvement of the hippocampus and parahippocampus (Figure 1A), regions with atrophy in our TLE group included middle and inferior temporal (Figure 1B), putamen and thalamus (Figure 1C) and supplementary motor areas (Figure 1D).

**Table 2:** Voxel-based morphometry of gray matter atrophy analysis

Brain Regions	x	y	z	T value	Z-score
Right thalamus	6	−20	8	4.27	4.02

Left thalamus	−2	−18	6	3.94	3.74

Left fusiform	−30	−38	−24	3.71	3.54

Right inferior temporal	58	−34	−24	3.11	3.00

Right fusiform	42	−16	−36	3.04	2.94

Right medial temporal	18	0	−36	2.95	2.85

Left caudate	−14	14	12	2.89	2.80

Left hippocampus	−18	−9	−20	2.84	2.76

Left putamen	−18	14	−6	2.40	2.35

Right supplementary motor area	6	−6	56	2.83	2.75

Right hippocampus	20	−6.24	−20	2.30	2.30

Left supplementary motor area	−4	−16	56	2.09	2.05

Right caudate nucleus	14	6	20	2.70	2.63

Left amygdala	−22	−6	−22	2.67	2.60

Right putamen	20	14	−4	2.38	2.33


[x,y,z] refers to Montreal Neurological Institute coordinates. T values were determined by dividing the estimated regression coefficient by its standard error

**Figure 2:**
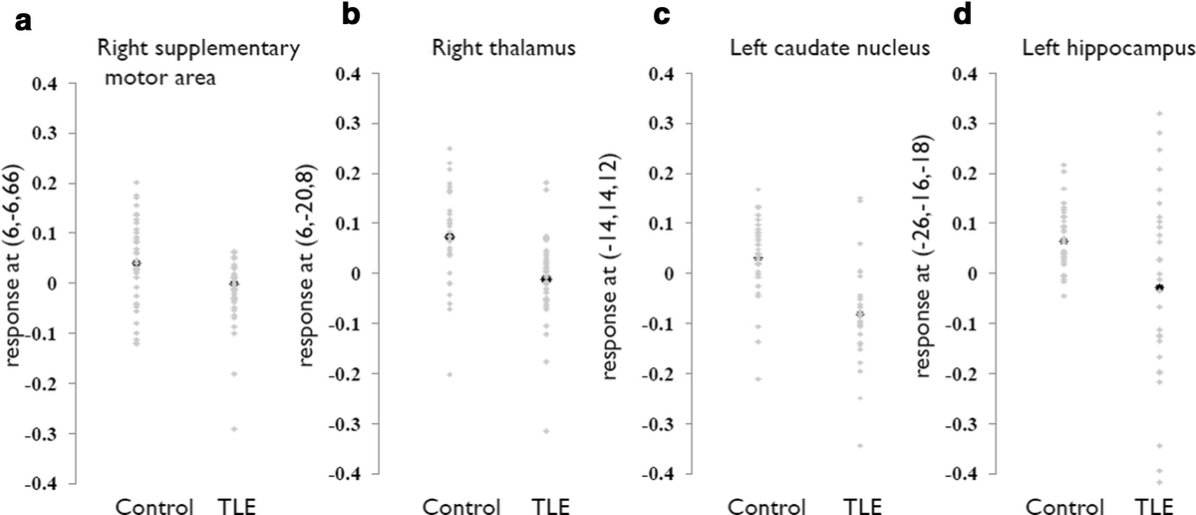
**Variability of fitted data of brain atrophy in the controls and patients with temporal lobe epilepsy (TLE)**. (x,y,z) refers to Montreal Neurological Institute coordinates.

Morphometric data with atrophic regions were extracted from four representative regions (Figure 1) to investigate the variability of voxel changes between the controls and patients with TLE (Figure 2). From the fitted plots, voxel variabilities from the right supplementary motor area (Figure 2A), right thalamus (Figure 2B) and left caudate nucleus (Figure 2C) in the patient group were small, and the values were all smaller than the controls. In contrast, the variability in the left hippocampus (Figure 2D) was large in the TLE group, although the mean voxel value was still smaller than the control group. Further, a higher level of HSP70 was correlated with a lower ipsilateral hippocampal volume after correcting for the effect of age in the linear regression model (R square = 0.22, *p *= 0.007).

### Clinical characteristics, biomarkers and neuropsychological tests results between Group 1 and 2

The TLE group was further divided into two groups according to seizure frequency as defined previously. Comparisons between the two patient groups in terms of biomarkers, neuropsychological performance and correlation with duration of epilepsy are shown in Table 3. Group 2 had significantly higher serum HSP70, NSE and S100ßP levels as compared with Group 1 (all *p *< 0.01), while the differences in plasma nuclear and mitochondrial DNA were not significant. Among the biomarkers, only the level of HSP70 had a positive correlation with duration of epilepsy (σ = 0.413, *p *< 0.01).

**Table 3:** Relationship of clinical characteristics with biomarkers and neuropsychiatric performances in patients with temporal lobe epilepsy

	Seizure frequency		Duration of epilepsy
	
	Group 1 (n = 22)	Group 2 (n = 12)	σ coefficient
Duration of epilepsy (years)	16.7 ± 8.5	20.3 ± 13.6	-
Number of antiepileptic drugs used Biomarkers	1.6 ± 0.6	2.52 ± 0.7	-0.029
Heat shock protein 70 (µg/ml)	123.4 ± 34.7	324.7 ± 97.4**	0.413†
Non-specific enolase (ng/ml)	8.4 ± 1.2	11.3 ± 1.13**	0.062
S100 ß protein (pg/ml)	79.04 ± 12.5	148.95 ± 43.6**	0.132
Plasma nuclear DNA (ng/ml)	29.22 ± 2.2	30.33 ± 2.2	0.18
Plasma mitochondrial DNA (ng/ml)	21.04 ± 1.0	21.13 ± 1.1	0.044
Verbal Memory CVLT-MS (9)			
30 sec free recall	7.86 ± 1.5	7.27 ± 2.8	0.041
10-min free recall	7.23 ± 1.9	7.09 ± 2.9	0.045
10-min recognition	7.50 ± 2.0	7.64 ± 1.6	0.153
Modified Rey-Osterrieth recall (17)	16.77 ± 0.8	16.82 ± 0.6	0.3020
Cube copy (2)	0.91 ± 0.9	1.18 ± 0.9	-0.068
Pentagon copy (1)	0.82 ± 0.4	0.82 ± 0.4	-0.469†
Semantic fluency animal (1 minute)	14.09 ± 4.5	17.73 ± 6.6	-0.342
Boston naming test (16)	14.36 ± 2.3	14.18 ± 2.4	-0.175
Comprehension (4)	3.45 ± 0.9	3.45 ± 1.0	-0.631†
Abstract thinking (3)	1.95 ± 1.2	2.27 ± 1.2	-0.347
Problem solving (3)	2.00 ± 1.0	2.00 ± 1.18	0.107
Stroop interference correct (1 minute)	39.82 ± 16.5	42.64 ± 12.4	-0.374
Design fluency	7.68 ± 5.7	9.00 ± 5.8	-0.350
Trail Making test time (< 120 seconds)	51.68 ± 38.8	45.18 ± 32.1	0.548†
Digit forward	7.27 ± 1.5	7.73 ± 1.1	-0.346

Values expressed as mean ± SD or with correlation coefficient (σ coefficient)

Neuropsychological tests listed above represent domains that were different from the control group; number in parenthesis following task name = maximal scores

*CVLT-MS *California Verbal Learning Test-Mental Status

Group 1 = seizure frequency ≤ 2 times per month; Group 2 = seizure frequency > 2 times per month

** *p *< 0.01 compared with Group 1 and Group 2, †*p *< 0.01 in correlation analysis

In neuropsychological performances, there were no significant differences between Group 1 and 2. However, with regards to the duration of epilepsy, an inverse correlation was found in pentagon copy and comprehension, while a positive correlation was found in Trail Making test completion time (*p *< 0.01).

## Discussion

The present study explored the clinical significance of serum biomarkers in relation to epileptic characteristics, cognitive performance and regions of GM atrophy patterns in patients with TLE. There were three major findings. First, the TLE group had higher serum levels of HSP70 and S100ßP and lower cognitive test scores compared with the controls. The serum levels of HSP70, NSE and S100ßP were significantly higher in the TLE patients defined by a higher seizure frequency (Group 2). Second, the inverse relationship between serum HSP70 levels with hippocampal GM partitions, memory dysfunction and duration of epilepsy suggest that HSP70 can be treated as a stress marker in TLE. Third, although the high variabilities in the hippocampal volume suggest the relatively lateralized nature of TLE, the homogenous atrophic patterns in regions beyond the epileptic origin (i.e. the supplementary motor area, thalamus and striatum) suggest more extensive structural damage in this epileptic disorder.

We found higher HSP70 levels in patients with TLE as compared with the controls. HSP70 belongs to the family of heat shock proteins that is involved in normal cellular homeostasis and survival by acting as a molecular chaperone [34]. It is a constitutive protein which is expressed after cell stress [6]. The role of HSP70 in patients with epilepsy is controversial. The induction of HSP70 in patients with TLE may reflect the stress condition conveyed by epileptic activities [17], however, a compensatory upregulation for neuroprotection has also been proposed [35]. In an animal model, overexpression of HSP70 due to status epilepticus had a positive correlation with seizure intensity, suggesting the role of HSP70 in reflecting the severity of neuronal damage [15]. Overexpression of HSP70 has similarly been observed in the hippocampal end folium neurons in patients with TLE, suggesting a correlation with the neuronal loss [16]. Although HSP70 expression in epilepsy may suggest stress rather than neuroprotection [17], induction of HSP70 in protecting hippocampal neurodegeneration via modulating endogenous glutamate expression has also been observed [35]. Therefore, the elevated serum levels of HSP70 can only be considered as a stress biomarker reflecting neuronal cell damage in the hippocampus, since an inverse relationship with memory test scores and hippocampal volume was found in addition to the elevation of HSP70 levels in patients with higher seizure frequencies and longer duration of epilepsy.

Another serum biomarker in this study showing a higher level in the patients with TLE as compared with the controls was S100ßP. The S100ßP homodimer is a neurite growth-promoting factor [36], and is widely used as a parameter for glial activation and blood brain barrier disruption [37]. In human tissues from patients with TLE, overexpression of S100ßP from astrocytes without a concurrent increase in glia fibrillary acidic protein suggests the pathophysiology of astrocyte dysregulation in TLE [13]. An elevated CSF level of S100ßP has been considered as a site-specific marker in patients with intractable TLE [38], also reflecting the severity of acute brain injury [39]. A significant elevation of serum S100ßP levels has been reported recently in Han Chinese TLE patients as compared to healthy controls, with a higher level especially in female patients [12]. Whether or not S100ßP is a suitable marker for epilepsy is still inconclusive. Some reports have indicated no differences in serum levels of S100ßP between patients with TLE and controls [11,40,41]. Taken together with the results of our study in that S100ßP was significantly higher in Group 2 patients, we suggest that the serum concentration of S100ßP could be another useful biomarker in patients with TLE for the evaluation of seizure activity.

The release of NSE from damaged neurons resulting from increased permeability of the blood brain barrier following seizure has been proposed as a possible mechanism [14]. In contrast with the uncertainties of S100ßP in epilepsy [40], the serum level of NSE is considered to be a more reliable marker for reflecting a longer duration of partial epilepsy [10]. Although elevated serum NSE levels have been frequently reported in patients with partial seizures or status epilepticus [11,14], the relationship with TLE was not found in a literature search. We did not detect a significant difference in NSE level between the TLE group and controls, however we did find higher serum NSE levels in the patients with a higher seizure frequency. Nevertheless, its role in relation to cognitive performance was not established in the present study nor was the correlation with duration of epilepsy in TLE patients. Compared with HSP70, the elevated S100ßP and NSE levels found in this study could be regarded as indirect evidence for blood brain barrier disruption.

Relatively little is known about circulating plasma nuclear and mitochondrial DNA in neurological diseases, however, they are generally considered to be markers of cell death in the form of necrosis or apoptosis [42]. The elevated plasma nuclear and mitochondrial DNA levels observed in critically ill patients [19] suggests a release of extracellular DNA from necrotic cells. Our study failed to detect differences either in plasma nuclear or mitochondrial DNA levels between the patients with TLE or controls. The inverse correlation of plasma nuclear DNA levels with executive function test may imply it is a neuronal damage marker, however further studies are needed to substantiate this explanation.

In TLE, there is a strong asymmetrical distribution of temporal lobe abnormalities with respect to epileptogenesis, particularly in the hippocampus and also to a lesser degree on the parahippocampal and entorhinal cortex [23]. In the present study, patients with TLE showed high lateralization of the epileptic focus from the initial EEG recording with ipsilateral hippocampal atrophy based on the morphometric data extracted from the hippocampus. It is worth pointing out that the correlation study between morphometric data with biomarkers was based on a hierachical order. We did not perform the correlation voxel-wisely to avoid the possibility of type I errors.

In contrast, in the extratemporal regions including the thalamus, supplementary motor area and caudate nucleus, the GM atrophy was relatively symmetric suggestive of common pathways of epileptogenesis through these structures. This observation may suggest the importance of kindling effects from the epileptic focus that either deactivate the inhibitory or stimulate the excitatory pathways and induce progressive damage to the related neuronal networks [34].

Although the study sample was small, we did not pool the patients into one in terms of epileptic lateralization for VBM analysis since the GM of the right and left cerebral hemispheres is asymmetric in healthy subjects, likely reflecting underlying functional specificity. The pathologic contribution from epilepsy to this asymmetry is substantially greater than normal underlying asymmetry. Since the study results were used to correlate with cognitive performances rather than tracing the epileptigenesis circuit, a general GM atrophic pattern of these patients was presented.

There are several limitations to this study. First, the patients were only enrolled if they had not received an operation for TLE, and we only selected patients without a history of mental retardation or psychiatric comorbidity. Whether the results of this study can only be applied to this unique epilepsy syndrome or whether they can be applied to the general population of epilepsy patients still requires larger study cohorts for verification. Second, long term AED therapy has been shown to contribute to atherosclerosis and oxidative stress in epilepsy patients [25]. As this study enrolled only a small number of patients and as the use of AEDs was not the same among all patients, the effects of the AEDs were therefore difficult to conclude. Different AEDs may have diverse impacts on the selected serum biomarkers levels included in this study. As such, interpretation of changes in biomarkers in the patients should be treated with great care. Lastly, the study design was mainly focused on the clinical parameters and neuroimaging findings with relation to the serum biomarkers during the interictal state. Therefore, the observations here can not represent the ictal pathogenesis of this disease model in relation with these biomarkers.

## Conclusions

The findings in the present study validated our initial hypothesis that biomarkers in the circulation may show predictive roles in the neuropsychological performance and neuroimaging changes in TLE. Serum levels of HSP70 had a positive correlation with the duration of epilepsy, hippocampal atrophy and memory dysfunction, suggesting that it can be used as a biomarker for stress-related neuronal damage. Moreover, compared with HSP70, the elevated S100ßP and NSE levels found in the present study may be regarded as indirect evidence for epileptic neuronal damage and blood brain barrier disruption. We are aware that the duration of epilepsy has been regarded as an important factor leading to the worsening of cognitive performance in patients with TLE [2] and this was also shown in the study results. The concurrent frontal-subcortical GM atrophy in patients with TLE reinforces the notion of a kindling phenomenon with neuronal damage from the related temporal foci in this highly lateralized epileptic disorder. A longitudinal study might extend the repertoire for disease progression while the evaluation in association with physiological parameters may unreveal the mechanism of epileptogenesis.

## Competing interests

The authors declare that they have no competing interests

## Authors' contributions

CCC, YCC designed the study, carried out the statistical analysis of the biomarkers and drafted the manuscript. CCL and CCL carried out the image acquisition, statistical analysis of the images and interpretation of the data. SDC, WNC, CHL, NCC carried out clinical data evaluation and statistical analysis. AYWC, SHHC conceived the study design and critically reviewed the manuscript. All authors have read and approved the final manuscript.

## Pre-publication history

The pre-publication history for this paper can be accessed here:

http://www.biomedcentral.com/1471-2377/12/15/prepub

## References

[B1] Engel J Jr A proposed diagnostic scheme for people with epileptic seizures and with epilepsy: report of the ILAE Task Force on Classification and Terminology Epilepsia 2001 42 6 796 803 10.1046/j.1528-1157.2001.10401.x 11422340

[B2] Engel J Jr Report of the ILAE classification core group Epilepsia 2006 47 9 1558 1568 10.1111/j.1528-1167.2006.00215.x 16981873

[B3] Thom M Mathern GW Cross JH Bertram EH Mesial temporal lobe epilepsy: How do we improve surgical outcome? Ann Neurol 2010 68 4 424 434 10.1002/ana.22142 20976764 PMC2966035

[B4] Tellez-Zenteno JF Hernandez Ronquillo L Moien-Afshari F Wiebe S Surgical outcomes in lesional and non-lesional epilepsy: a systematic review and meta-analysis Epilepsy Res 2010 89 2-3 310 318 10.1016/j.eplepsyres.2010.02.007 20227852

[B5] Friedman DE Gilliam FG Seizure-related injuries are underreported in pharmacoresistant localization-related epilepsy Epilepsia 2010 51 1 43 47 10.1111/j.1528-1167.2009.02170.x 19519796

[B6] Chen DK So YT Fisher RS Use of serum prolactin in diagnosing epileptic seizures: report of the Therapeutics and Technology Assessment Subcommittee of the American Academy of Neurology Neurol 2005 65 5 668 675 10.1212/01.wnl.0000178391.96957.d0 16157897

[B7] Cui J Wang Y Dong Q Wu S Xiao X Hu J Chai Z Zhang Y Morphine protects against intracellular amyloid toxicity by inducing estradiol release and upregulation of Hsp70 J Neurosci 2011 31 45 16227 16240 10.1523/JNEUROSCI.3915-11.2011 22072674 PMC6633253

[B8] Rabinowicz AL Correale JD Bracht KA Smith TD DeGiorgio CM Neuron-specific enolase is increased after nonconvulsive status epilepticus Epilepsia 1995 36 5 475 479 10.1111/j.1528-1157.1995.tb00489.x 7614925

[B9] Thom M Seetah S Sisodiya S Koepp M Scaravilli F Sudden and unexpected death in epilepsy (SUDEP): evidence of acute neuronal injury using HSP-70 and c-Jun immunohistochemistry Neuropathol Appl Neurobiol 2003 29 2 132 143 10.1046/j.1365-2990.2003.00452.x 12662321

[B10] DeGiorgio CM Correale JD Gott PS Ginsburg DL Bracht KA Smith T Boutros R Loskota WJ Rabinowicz AL Serum neuron-specific enolase in human status epilepticus Neurol 1995 45 6 1134 1137 10.1212/wnl.45.6.1134 7783877

[B11] Buttner T Lack B Jager M Wunsche W Kuhn W Muller T Przuntek H Postert T Serum levels of neuron-specific enolase and s-100 protein after single tonic-clonic seizures J Neurol 1999 246 6 459 461 10.1007/s004150050383 10431771

[B12] Lu C Li J Sun W Feng L Li L Liu A Mao W Wei H Gao L Zhang X Elevated plasma S100B concentration is associated with mesial temporal lobe epilepsy in Han Chinese: a case-control study Neurosci Lett 2010 484 2 139 142 10.1016/j.neulet.2010.08.036 20727940

[B13] Griffin WS Yeralan O Sheng JG Boop FA Mrak RE Rovnaghi CR Burnett BA Feoktistova A Van Eldik LJ Overexpression of the neurotrophic cytokine S100 beta in human temporal lobe epilepsy J Neurochem 1995 65 1 228 233 7790864 10.1046/j.1471-4159.1995.65010228.x PMC3833622

[B14] Palmio J Keranen T Alapirtti T Hulkkonen J Makinen R Holm P Suhonen J Peltola J Elevated serum neuron-specific enolase in patients with temporal lobe epilepsy: a video-EEG study Epilepsy Res 2008 81 2-3 155 160 10.1016/j.eplepsyres.2008.05.006 18595663

[B15] Hashimoto K Watanabe K Nishimura T Iyo M Shirayama Y Minabe Y Behavioral changes and expression of heat shock protein hsp-70 mRNA, brain-derived neurotrophic factor mRNA, and cyclooxygenase-2 mRNA in rat brain following seizures induced by systemic administration of kainic acid Brain Res 1998 804 2 212 223 10.1016/S0006-8993(98)00708-2 9757041

[B16] Ryufuku M Toyoshima Y Kitaura H Zheng Y Fu YJ Miyahara H Murakami H Masuda H Kameyama S Takahashi H Hypertrophy of hippocampal end folium neurons in patients with mesial temporal lobe epilepsy Neuropathology 2011 31 5 476 485 10.1111/j.1440-1789.2010.01191.x 21276083

[B17] Yang T Hsu C Liao W Chuang JS Heat shock protein 70 expression in epilepsy suggests stress rather than protection Acta Neuropathol 2008 115 2 219 230 10.1007/s00401-007-0297-3 17929041

[B18] Tsai NW Lin TK Chen SD Chang WN Wang HC Yang TM Lin YJ Jan CR Huang CR Liou CW The value of serial plasma nuclear and mitochondrial DNA levels in patients with acute ischemic stroke Clin Chim Acta 2011 412 5-6 476 479 10.1016/j.cca.2010.11.036 21130757

[B19] Lu CH Chang WN Tsai NW Chuang YC Huang CR Wang HC The value of serial plasma nuclear and mitochondrial DNA levels in adult community-acquired bacterial meningitis QJM 2010 103 3 169 175 10.1093/qjmed/hcp201 20129945

[B20] Pitkanen A Sutula TP Is epilepsy a progressive disorder? Prospects for new therapeutic approaches in temporal-lobe epilepsy Lancet Neurol 2002 1 3 173 181 10.1016/S1474-4422(02)00073-X 12849486

[B21] Andersson-Roswall L Engman E Samuelsson H Malmgren K Cognitive outcome 10 years after temporal lobe epilepsy surgery: a prospective controlled study Neurol 2010 74 24 1977 1985 10.1212/WNL.0b013e3181e39684 20548042

[B22] Baxendale S Heaney D Thompson PJ Duncan JS Cognitive consequences of childhood-onset temporal lobe epilepsy across the adult lifespan Neurol 2010 75 8 705 711 10.1212/WNL.0b013e3181eee3f0 20733146

[B23] Keller SS Roberts N Voxel-based morphometry of temporal lobe epilepsy: an introduction and review of the literature Epilepsia 2008 49 5 741 757 10.1111/j.1528-1167.2007.01485.x 18177358

[B24] Yang T Zhou D Stefan H Why mesial temporal lobe epilepsy with hippocampal sclerosis is progressive: uncontrolled inflammation drives disease progression? J Neurol Sci 2010 296 1-2 1 6 10.1016/j.jns.2010.06.002 20663517

[B25] Tan TY Lu CH Chuang HY Lin TK Liou CW Chang WN Chuang YC Long-term antiepileptic drug therapy contributes to the acceleration of atherosclerosis Epilepsia 2009 50 6 1579 1586 10.1111/j.1528-1167.2009.02024.x 19292757

[B26] Lo YM Tein MS Lau TK Haines CJ Leung TN Poon PM Wainscoat JS Johnson PJ Chang AM Hjelm NM Quantitative analysis of fetal DNA in maternal plasma and serum: implications for noninvasive prenatal diagnosis Am J Hum Genet 1998 62 4 768 775 10.1086/301800 9529358 PMC1377040

[B27] Chiu RW Chan LY Lam NY Tsui NB Ng EK Rainer TH Lo YM Quantitative analysis of circulating mitochondrial DNA in plasma Clin Chem 2003 49 5 719 726 10.1373/49.5.719 12709361

[B28] Pfaffl MW A new mathematical model for relative quantification in real-time RT-PCR Nucleic Acids Res 2001 29 9 e45 10.1093/nar/29.9.e45 11328886 PMC55695

[B29] Chang CC Chang WN Lui CC Wang JJ Chen CF Lee YC Chen SS Lin YT Huang CW Chen C Longitudinal study of carbon monoxide intoxication by diffusion tensor imaging with neuropsychiatric correlation J Psychiatry Neurosci 2010 35 2 115 125 10.1503/jpn.090057 20184809 PMC2834793

[B30] Chang CC Chang YY Chang WN Lee YC Wang YL Lui CC Huang CW Liu WL Cognitive deficits in multiple system atrophy correlate with frontal atrophy and disease duration Eur J Neurol 2009 16 10 1144 1150 10.1111/j.1468-1331.2009.02661.x 19486137

[B31] Chang CC Lee YC Chang WN Chen SS Lui CC Chang HW Liu WL Wang YL Damage of white matter tract correlated with neuropsychological deficits in carbon monoxide intoxication after hyperbaric oxygen therapy J Neurotrauma 2009 26 8 1263 1270 10.1089/neu.2008.0619 19317622

[B32] Chang CC Kramer JH Lin KN Chang WN Wang YL Huang CW Lin YT Chen C Wang PN Validating the Chinese version of the Verbal Learning Test for screening Alzheimer's disease J Int Neuropsychol Soc 2010 16 2 244 251 10.1017/S1355617709991184 20003579 PMC3767760

[B33] Ashburner J Friston KJ Voxel-based morphometry-the methods NeuroImage 2000 11 6 Pt 1 805 821 10860804 10.1006/nimg.2000.0582

[B34] Lanneau D Wettstein G Bonniaud P Garrido C Heat shock proteins: cell protection through protein triage ScientificWorldJournal 2010 10 1543 1552 20694452 10.1100/tsw.2010.152 PMC5763791

[B35] Ayala GX Tapia R HSP70 expression protects against hippocampal neurodegeneration induced by endogenous glutamate in vivo Neuropharmacol 2008 55 8 1383 1390 10.1016/j.neuropharm.2008.08.035 18789952

[B36] Marshak DR S100 beta as a neurotrophic factor Prog Brain Res 1990 86 169 181 2087556

[B37] Pham N Fazio V Cucullo L Teng Q Biberthaler P Bazarian JJ Janigro D Extracranial sources of S100B do not affect serum levels PLoS One 2010 5 9 e12691. doi:10.1371/journal.pone.0012691 10.1371/journal.pone.0012691 PMC2937027 20844757

[B38] Steinhoff BJ Tumani H Otto M Mursch K Wiltfang J Herrendorf G Bittermann HJ Felgenhauer K Paulus W Markakis E Cisternal S100 protein and neuron-specific enolase are elevated and site-specific markers in intractable temporal lobe epilepsy Epilepsy Res 1999 36 1 75 82 10.1016/S0920-1211(99)00026-1 10463853

[B39] Kleindienst A Meissner S Eyupoglu IY Parsch H Schmidt C Buchfelder M Dynamics of S100B release into serum and cerebrospinal fluid following acute brain injury Acta Neurochir Suppl 2010 106 247 250 10.1007/978-3-211-98811-4_46 19812958

[B40] Leutmezer F Wagner O Baumgartner C Serum s-100 protein is not a suitable seizure marker in temporal lobe epilepsy Epilepsia 2002 43 10 1172 1174 10.1046/j.1528-1157.2002.50101.x 12366732

[B41] Palmio J Peltola J Vuorinen P Laine S Suhonen J Keranen T Normal CSF neuron-specific enolase and S-100 protein levels in patients with recent non-complicated tonic-clonic seizures J Neurol Sci 2001 183 1 27 31 10.1016/S0022-510X(00)00478-0 11166790

[B42] Fournie GJ Martres F Pourrat JP Alary C Rumeau M Plasma DNA as cell death marker in elderly patients Gerontol 1993 39 4 215 221 10.1159/000213536 8244049

